# Report of a Case of Cholera Infantum

**Published:** 1866-10

**Authors:** H. L. Wilson

**Affiliations:** Atlanta, Georgia


### ARTICLE IV.

*Report of a Case of Cholera Infantum.* By H. L. Wilson, M. D., Atlanta, Georgia.

September 5th, 1866. I was called to see a child, æt. 8 months, who had been spending a month or six weeks in South-western Georgia. The child had been fretful for several days. The mother had been very much reduced from intermittent fever, thereby diminishing the quantity of nourishment previously given, which was mentioned as the cause of the child's illness. Upon inquiry I found that the little patient was having frequent alvine discharges, and prescribed chalk mixture. I was sent for again about two o'clock. The defecations were growing more frequent. I made the following prescription:

Aromat. Syrp. Rhei, ʒ viii.

Camph. Tinct. Opii, ʒ iv.

Aqua Ammonia, ʒ i.

Mix.

Tea-spoonful every two hours until relieved.

I saw the babe again at nightfall; no improvement whatever. I gave strict attention during the entire night; applied warm fomentations to the bowels; gave starch and tinct. opii clysters; gave during the night at least twenty drops tinct. opii. At day, on the morning of the 6th, there was but little improvement. At nine o'clock, just twenty-four hours from its diarrhœal attack, it had averaged three evacuations per hour. During the day and night I used the above prescription, together with brandy and mucilage per orem. Also catechu and starch enemas. From 9 o'clock September 6th to 9 September 7th, the evacuations averaged two per hour, with intense griping. To-day, prescribed:

Pulv. Doveri, gr. iii.

" Camph., gr. i.

Hydrarg. Chlo. Mite, gr. i.

This to be given every three hours.

After taking the first one, he slept soundly: the skin was less hot and harsh; pulse fuller; bowels moved less frequently—only having about one action per hour during the day and night. Vomiting ensued, however, to such an extent that I was compelled to stop the use of the powders. The mother was even forced to cease nursing. I applied bran poultices, sprinkled with mustard, to the entire abdomen; during the night, the vomiting ceased. The following enema was administered:

Acetas Plumbi, gr. xv.
Mucilage gum acacia, ℥ ii.

It produced no impression whatever. I then used by injection:

Nitrate Silver, gr. v.
Mucilage gum acacia, ℥ iv.

It produced a little more griping, but failed to check the bowels, the evacuations continuing, one an hour, during the day and night.

Sept. 8.—This morning at daylight I prescribed:

Sulph. morph., 1-18 gr.
Sub. nit. bismuth, iv. gr.

The child was restless; pulse small and frequent; skin harsh, hot and burning to the touch. Ordered warmth to the abdomen. Saw him at noon. Had slept well; stretched out full length; no movements of the bowels; full inspirations; pulse full and less frequent. Saw the child about dark; bowels had been moved only three times; abdomen flabby; no pain upon pressure, but growing fretful. I repeated the last prescription with the same delightful effect, till 11 o'clock at night, when the mother, fearing to repeat the dose, the evacuations became again frequent.

Sept. 9.—Saw the child very early. Gave more morphine and bismuth. Ordered weak sangaree to be given *pro re nata*. Saw the child at one o'clock; bowels quiet, apparently better. Saw him again at sundown; had five actions during the afternoon; found him fretting from prolapsus ani. Ordered the parts bathed freely with cold water.

Sept. 10.—Saw little patient at 8 o'clock. Had one evacuation per hour during the night—the last action containing blood and mucus, differing from the previous green and watery actions. I ordered mustard and mush to the bowels, and

Hydr. chlo. mite, gr. ii.
Four powders, one every hour.
Also morphine and bismuth.

About ten o'clock he had another bloody action. Saw him at dark; found him quietly sleeping; pulse full and almost natural; skin pleasant; head cool. While there, he had an action evidently from the medicine; no blood; no griping—has been no vomiting during the day. Advised the mother to allow the child to nurse but little at a time. Urged the necessity of great care in *her* diet.

Sept. 11.—Saw the child early in the morning; found him asleep. Had one action during the night. Ordered the morphine and bismuth to be continued as previously prescribed, increasing the dose of the former to 1-10 of a grain. Also ordered weak toddy, tea-spoonful every two hours. Called in the morning late; found my little patient in deep sleep from the medicine; bowels had been moved only three times since my visit in the morning. The cold application had relieved the prolapsus. He was less fretful.

Sept. 12.—Saw my patient at nine o'clock. He slept pretty well, having only one action during the night. While present, he had two evacuations, one blood and mucus. I gave two grains of calomel to be taken at bedtime. Had prescribed acetas plumbi in the morning, but the mother thought it produced emesis.

Sept. 13.—The child had one action during the night; no blood; no pain; is disposed to play; notices more. I ordered the continuance of morphine, according to circumstances; also warm bath at night; continued the sangaree or toddy.

Sept. 14.—Saw my little patient early. Found him playing with his toys. Pulse good; moist skin; actions more natural.

Sept. 15.—My patient still improves; convalescing rapidly.

Sept. 16.—Saw the child playing on the floor; cheerful and eyes bright. Discharged the case.

